# Effect of Farnesol in *Trichoderma* Physiology and in Fungal–Plant Interaction

**DOI:** 10.3390/jof8121266

**Published:** 2022-11-30

**Authors:** Rosa E. Cardoza, Susan P. McCormick, Laura Lindo, Sara Mayo-Prieto, David González-Cazón, Natalia Martínez-Reyes, Guzmán Carro-Huerga, Álvaro Rodríguez-González, Robert H. Proctor, Pedro A. Casquero, Santiago Gutiérrez

**Affiliations:** 1 University Group for Research in Engineering and Sustainable Agriculture (GUIIAS), Area of Microbiology, Universidad de León, 24400 Ponferrada, Spain; 2 Mycotoxin Prevention and Applied Microbiology Research Unit, National Center for Agricultural Utilization Research, Agriculture Research Service, U.S. Department of Agriculture, Peoria, IL 61604, USA; 3 University Group for Research in Engineering and Sustainable Agriculture (GUIIAS), Area of Plant Production, Universidad de León, 24009 León, Spain

**Keywords:** *Trichoderma*, farnesol, terpene biosynthesis, gene overexpression, *Trichoderma*-plant interaction, plant-defense related genes

## Abstract

Farnesol is an isoprenoid intermediate in the mevalonate (MVA) pathway and is produced by the dephosphorylation of farnesyl diphosphate. Farnesol plays a central role in cell growth and differentiation, controls production of ubiquinone and ergosterol, and participates in the regulation of filamentation and biofilm formation. Despite these important functions, studies of farnesol in filamentous fungi are limited, and information on its effects on antifungal and/or biocontrol activity is scarce. In the present article, we identified the *Trichoderma harzianum* gene *dpp1*, encoding a diacylglycerol pyrophosphatase that catalyzes production of farnesol from farnesol diphosphate. We analyzed the function of *dpp1* to address the importance of farnesol in *Trichoderma* physiology and ecology. Overexpression of *dpp1* in *T. harzianum* caused an expected increase in farnesol production as well as a marked change in squalene and ergosterol levels, but overexpression did not affect antifungal activity. In interaction with plants, a *dpp1*-overexpressing transformant acted as a sensitizing agent in that it up-regulated expression of plant defense salicylate-related genes in the presence of a fungal plant pathogen. In addition, toxicity of farnesol on *Trichoderma* and plants was examined. Finally, a phylogenetic study of *dpp1* was performed to understand its evolutionary history as a primary metabolite gene. This article represents a step forward in the acquisition of knowledge on the role of farnesol in fungal physiology and in fungus-environment interactions.

## 1. Introduction

Terpenes are the most abundant secondary metabolites in nature and have a wide variety of physiological functions. *Trichoderma* species produce a number of these compounds [1,2]. Up to now, several genes involved in their biosynthesis have been characterized and their biological functions described. Among them, the genes belonging to the ergosterol biosynthetic pathway, e.g., *erg1*, *erg7* and *erg9*, are the best characterized [3,4,5].

Farnesyl diphosphate (FDP) is an essential intermediate in biosynthesis of terpenes and thereby plays a central role in terpene metabolism. FDP acts a primary metabolite that participates in the prenylation of proteins involved in the regulation of cell growth, proliferation, cytoskeletal reorganization, and in the trafficking of intracellular membrane vesicles, which are essential to ensure proper cellular function [6]. In addition, the level of FDP is also related with the production of farnesol, which regulates important processes as fungal filamentation [7], formation of biofilms, or quorum sensing responses, acting in these functions as a tyrosol-antagonistic molecule [8]. Farnesol itself also exhibits a toxic effect against yeast and filamentous fungi [9]. Thus, the level of FDP must be tightly regulated. One of the steps that has been little studied is the dephosphorylation of FDP to give rise to farnesol, which is likely catalyzed by diphosphate phosphatases. Two diacylglycerol-P-P phosphatases have been identified in *Saccharomyces cerevisiae* (Lpp1p and Dpp1p) (GenBank accession numbers NM_001180811 and NM_001180592, respectively) [10,11,12] and *Candida albicans* (Dpp2 and Dpp3). These enzymes participate in the dephosphorylation of a variety of diphosphate compounds, including diphosphate derivatives of dolichyl, dolichyl, farnesyl, and geranylgeranyl [13]. Thus, they are candidates to catalyze FDP dephosphorylation to give rise to farnesol.

**Figure 1:**
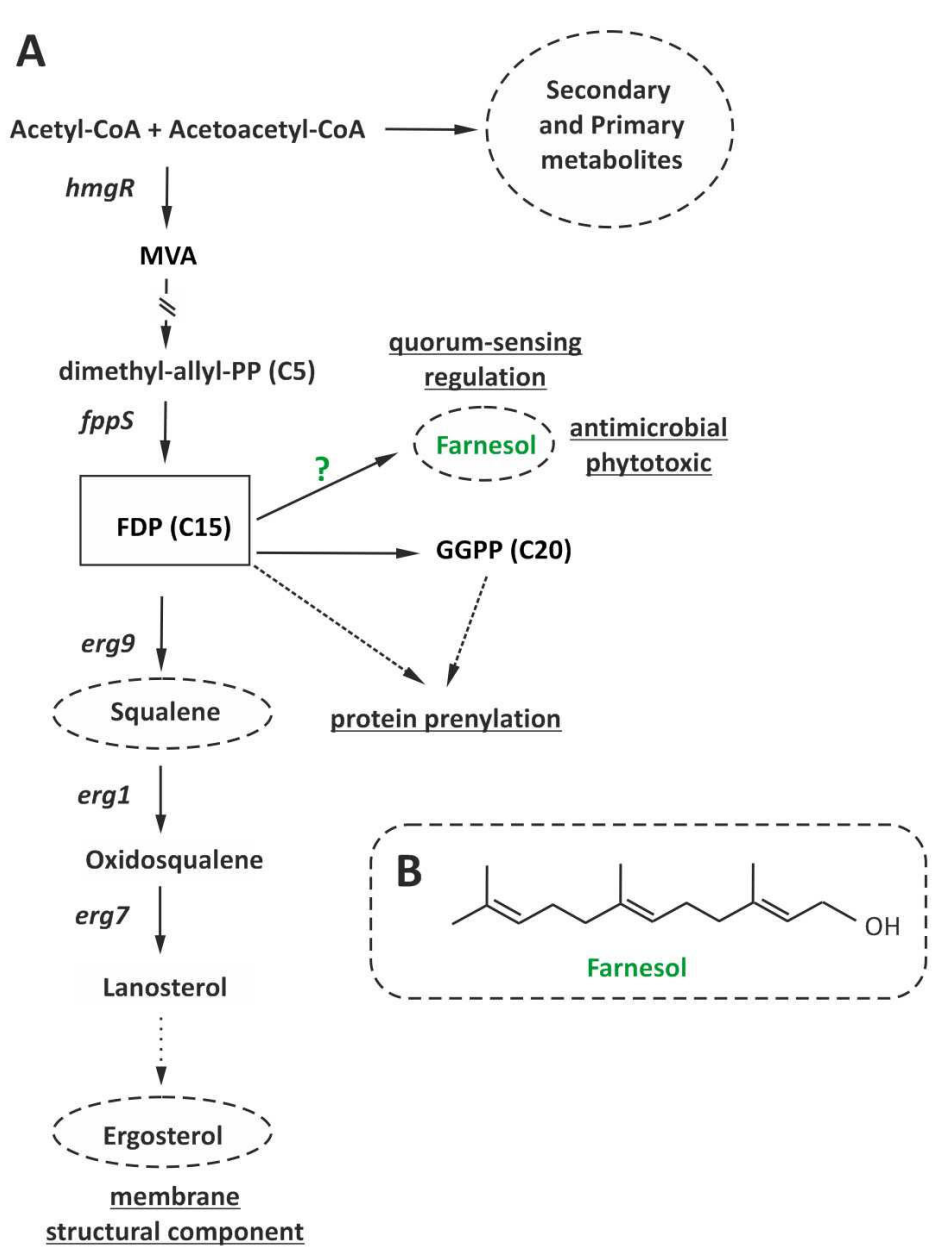
(**A**) Schematic representation of the ergosterol biosynthetic pathway, pointing out the genes analyzed in this study. FDP, farnesyl diphosphate; GGPP, geranyl geranyl pyrophosphate; MVA, mevalonic acid; *hmgR*, *fppS*, *erg9*, *erg1*, and *erg7*, correspond to genes encoding for hydroxymethylglutaryl-CoA reductase, farnesyl diphosphate synthase, squalene synthase, squalene epoxidase, and oxidosqualene cyclase, respectively. A green question mark (?) was included to indicate the gene involved in FDP dephosphorylation whose identification is the main objective of the current study. (**B**) Chemical structure of farnesol.

To our knowledge, no data on the function of *DPP* or *LPP* homologs in *Trichoderma* species or other filamentous fungi have been reported. However, in previous studies on biosynthesis of the sesquiterpenoid mycotoxins trichothecenes in *Trichoderma* species, whose biosynthesis requires FDP as a precursor, it was shown that mutants reduced in their ability to produce the trichothecene harzianum A (HA); surprisingly, in addition, they produced lower levels of farnesol [14] when media were supplemented with terbinafine (TRB), an allylamine antifungal drug that disrupts the normal sterol biosynthetic pathway, by specifically inhibiting the squalene epoxidase (ERG1) enzyme (Figure 1) [3,15]. These data indicate that blocking the channeling of FDP to the ergosterol pathway by the inhibitory activity of TRB or toward HA in the mutants used in that study resulted in an increase in farnesol production by fungal cells as a way to avoid FDP accumulation. By contrast, exposure to TRB increased HA production in wild-type *T. arundinaceum* without affecting farnesol levels [14]. These results provide evidence that the HA biosynthetic pathway contributes to the maintenance of homeostasis of the terpenoid biosynthetic network. An increase in the level of farnesol was also observed in wild-type *C. albicans* in response to zaragozic acid B, which also inhibits squalene epoxidase [16]. The increases in farnesol accumulation observed in the latter suggest that that *C. albicans* does not have an alternative pathway to redirect FDP that accumulates as a result of squalene epoxidase inhibition, which would notably influence its pathogenic properties. In fact, *C. albicans* treated with fluconazole, a triazole analog that inhibits the sterol 14α-demethylase, which is involved in the demethylation of lanosterol to give rise to 4,4-dimethyl cholesta 8,14,24-trienol, an early committed step in ergosterol biosynthesis, drastically increased their level of farnesol production, further increasing *Candida* virulence [17].

In the current study, we assessed the importance of farnesol in *Trichoderma* physiology and ecology by first identifying the *Trichoderma harzianum* homologs (*dpp1*/*lpp1*) to the *S. cerevisiae DPP1* and *LPP1* genes. We subsequently determined the effects of overexpression of *dpp1* in *T. harzianum* on farnesol production, antifungal activity, ergosterol and squalene levels, and in the ability of the fungus to colonize tomato roots and to regulate tomato-defense-related genes. Finally, we examined the relationships of *dpp1* orthologs from species representing the breadth of phylogenetic diversity of the genus *Trichoderma*.

## 2. Materials and Methods

### 2.1. Strains Used and Culture Conditions

*Trichoderma harzianum* CECT 2413 (=T34) was used as a recipient in *dpp1* gene overexpression experiments. *Trichoderma* strains were maintained and sporulated on potato dextrose agar (PDA) medium (Becton Dickinson Co, Sparks, MA, USA). To induce sporulation, strains were incubated at 28 °C for 4–7 days in the dark. *Botrytis cinerea* strain B05.10 was used in the antifungal and in plant infection assays. Sporulation of this strain was induced by growing it on V8-juice agar medium (2% agar) (Oxoid Ltd., Basingstoke, UK) for 7 days at 21 °C and a photoperiod of 16 h light/8 h dark. *Rhizoctonia solani* strain R43 was also used in antifungal assays and was grown on PDA medium at 28 °C for 5–7 days in the dark.

Five additional growth media were used to study the phenotypic differences between the *Trichoderma* strains characterized in the present study, including oatmeal agar (OA) medium (1 L oat meal extract, 1.5% agar), cornmeal agar (CMA) medium (0.1% yellow cornmeal, 0.5% potato dextrose broth, 1.5% agar), synthetic nutrient-poor agar (SNA) medium (0.1% KH_2_PO_4_, 0.1% KNO_3_, 0.05% MgSO_4_·7H_2_O, 0.05% KCl, 0.02% glucose, 0.02% sucrose, 1.5% agar), and Mung bean broth agar (MBA) medium (1 L Mung bean tea, 2% agar).

*Trichoderma* strains generated during the current study and the *Trichoderma* control strain (T34-43b1.3) were also grown in liquid *Trichoderma* minimal medium (MMT) [18] to determine the effect of exogenous farnesol on production of ergosterol, squalene, and in the level of expression of genes related to the terpene biosynthetic pathway.

### 2.2. Overexpression of dpp1 Gene in T. harzianum CECT 2413 (T34)

**Figure 2:**
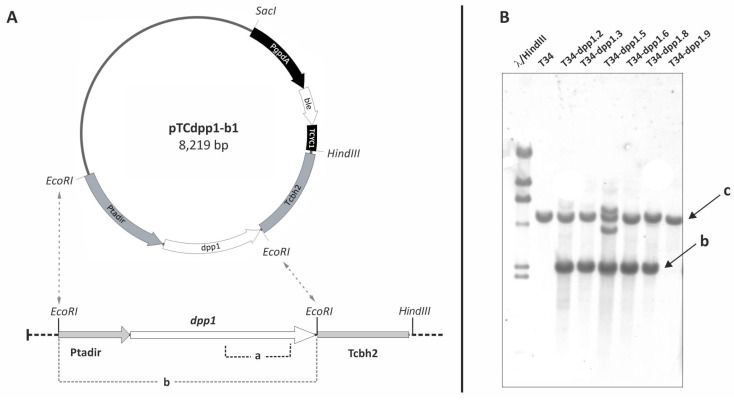
(**A**) Schematic representation of pTCdpp1-b1. PgpdA, promoter of the *Aspergillus nidulans gpdA* gene; *ble*, phleomycin resistance gene from *Streptoalloteichus hindustanus*; TCYC1, transcriptional terminator of the *Saccharomyces cerevisiae* cytochrome C oxidase gene; *dpp1*, *T. harzianum dpp1* gene. Ptadir, promoter of the *T. harzianum tss1* gene, and Tcbh2, transcriptional terminator of the *T. reesei* cellobiohydrolase 2 encoding gene. (**B**) Southern blot analysis of genomic DNAs from T34 (control) and six transformants with plasmid pTCdpp1-b1, digested with *Eco*RI, and hybridized with the 622 bp probe (a) indicated in panel A. Note that in T34 and T34-dpp1.9 (included as a negative control in the Southern), we only observed the wild-type 4.910 bp band (c), corresponding to the endogenous copy of the *dpp1* gene. (b) 2341 bp *Eco*RI fragment expected for the *dpp1*-overexpression transformants in the southern experiments.

Construction of pTCdpp1-b1. The homolog to *DPP1/LPP1* gene (*dpp1*) identified in the genome sequence of *T. harzianum* CBS226.95 v1.0 (Join Genome Institute: jgi.doe.gov/ (accessed on 8 November 2022)) was PCR amplified with the Pfu polymerase (EURx, Gdansk, Poland), using as template the genomic DNA of the *T. harzianum* T34 strain, and primers Th-dpp1 5F/Th-dpp1 3R (Table S1). The resulting amplified fragment (1137 bp) was phosphorylated with the polynucleotide kinase (Thermo Fisher, Waltham, MA, USA), and, after gel purification, the fragment was ligated to the pBluescript KS+ plasmid (Stratagene, La Jolla, CA, USA) linearized with *Eco*RV and dephosphorylated. The resulting plasmid, pKS_dpp1 (4098 bp), was digested with endonucleases *Hin*dIII-*Eco*RI, to isolate the *dpp1*-gene fragment, which was then treated with Klenow, gel purified, and ligated to pTAcbh2 [19] previously digested with *Nco*I, filled with Klenow fragment (Thermo Fisher) and dephosphorylated with CIAP (Fermentas). The resulting plasmid pTCdpp1 (6495 bp) was digested with *Bgl*II-*Hin*dIII to isolate the 3723 bp fragment containing the Th-*dpp1* overexpression cassette, filled with Klenow fragment, and ligated to the plasmid pJL43b1 [20], previously digested with *Acc*65I and treated with Klenow to obtain the plasmid pTCdpp1-b1 (8219 bp) (Figure 2A). Finally, this plasmid was linearized with *Hin*dIII prior to its transformation into *T. harzianum* protoplasts.

### 2.3. Transformation of pTCdpp1-b1 Plasmid in T. harzianum T34

Plasmid pTCdpp1-b1 was linearized with *Hin*dIII and transformed in the *T. harzianum* T34 strain following a protoplast-based procedure [20], but using Czapek as regeneration medium (sucrose, 30 g/L; NaNO_3_, 2 g/L; K_2_HPO_4_, 0.5 g/L; MgSO_4_·7H_2_O, 0.5 g/L; FeSO_4_·7H_2_O, 0.01 g/L; Agar 1.5%) supplemented with 1 M sorbitol as osmotic stabilizer and 100 μg/mL phleomycin as selection marker.

### 2.4. DNA Probes Labeling and Southern Hybridization

Genomic DNA purification, labeling of DNA probes, and southern hybridization experiments were performed as previously described [20].

### 2.5. Real-Time qPCR (qPCR) Analysis

Reactions were carried out as described previously [21]. Expression ratios and values of statistical significance were calculated using REST^© 2022^ software [22], using the α-actin as a housekeeping internal control gene, for both *Trichoderma* and tomato gene expression analysis. Each sample was analyzed in triplicate.

In *Trichoderma* strains, expression of genes *hmgR*, encoding the hydroxymethylglutaryl-CoA reductase; *erg1*, encoding the squalene epoxidase; *erg7*, encoding the oxidosqualene-lanosterol cyclase; *erg9*, encoding squalene synthase; and *dpp1*, characterized in the present study, all involved to the terpene pathway, and additionally *laeA*, *veA* and *velB*, belonging to the regulatory VELVET complex, was analyzed by qPCR.

In the tomato qPCR studies, four different groups of genes were analyzed: (i) genes involved in the salicylate (SA) signaling pathway, including *PR1b1,* and *PR-P2* [23]; (ii) genes belonging to the jasmonate/ethylene (JA/ET) signaling pathways, including *PINI*, *PINII*, *TomLoxA* [23]; *ACCS* that encodes the enzyme ACC (1-aminocyclopropane 1-carboxylate), the latter catalyzing the synthesis of ACC in the ethylene (a plant hormone) biosynthetic pathway as a derived compound of the methionine cycle; and (iii) genes involved in growth and development, including *SUCS* (encoding sucrose synthase, which is related to plant growth) and *GAI*, the product of which is a DELLA protein growth repressor (a plant development-related gene) [21,24].

Oligonucleotide sequences and PCR amplification efficiencies are included in Table S1.

### 2.6. Metabolomic Characterization

For this study, liquid cultures were filtered through Miracloth^®^ (Millipore Co., Billerica, MA, USA) sterile filters, and both the mycelia and the culture filtrates were used for further analyses.

#### 2.6.1. Quantification of Farnesol

Levels of intra- and extracellular cellular farnesol were quantified following an HPLC procedure. This compound was quantified from both culture broths (=culture filtrates) and mycelia through the following steps. (i) Extraction of farnesol from culture broths: First, 2 mL of broth were extracted with 400 μL of ethyl acetate, mixed and centrifuged for 10 min at 4000 rpm. Supernatant was collected and extracted twice again following the same procedure. Finally, the extract was evaporated in a SpeedVac vacuum concentrator (Thermo Fisher) and resuspended in 50 μL acetonitrile. (ii) Extraction of farnesol from mycelia: Mycelia were frozen at −80 °C, freeze-dried, and pestled to powder in a mortar. Mycelia (50 mg each) were resuspended into 1 mL of Tris-HCl 50 mM, pH 6.8 buffer, extracted with 200 μL of ethyl acetate and processed as described above. (iii) HPLC quantification: A measure of 20 μL of the concentrated extracts was applied to a reversed-phase C18 column of 150 mm × 4.6 mm i.d., 4 μm (octadecyl-silane (ODS)) (YMC Europe GmbH) and eluted with a 1 mL/min flux of mobile phase (90% water/10% acetonitrile at zero time, performing a gradient to reach 100% acetonitrile in 40 min, then returned to 10% acetonitrile at 50 min). Using these conditions, farnesol eluted at 34.7 min and quantification was carried out by comparison of the peak area with a standard curve. For determination of the specific production of farnesol, the total protein concentration from culture supernatants was quantified as described by Bradford (1976) [25].

#### 2.6.2. Quantification of Ergosterol and Squalene

Total intracellular sterols were extracted from the mycelia harvested from the same cultures used for HA quantification. Ergosterol and squalene contents were calculated as reported previously [4,26].

### 2.7. Antifungal Assays

#### 2.7.1. Antifungal Assays on Tomato Leaves

Antifungal assays were made on leaves detached from 4-week-old tomato plants. Leaves were first inoculated with a suspension of *B. cinerea* B05.10 spores (5 × 10^5^ spores/mL) placed in two spots/leaf. Once the spots were dried, 15 μL of 96 h PDB broths collected from the *Trichoderma* strains analyzed were placed over each spot. Leaves were incubated for four days, and the lesion diameters were measured.

#### 2.7.2. Antifungal Assays on Membranes against *Rhizoctonia solani*

The ability of *dpp1*-overexpressed transformants to inhibit growth of the fungal phytopathogen *R. solani* R43 was assessed by a membrane-based approach that was performed as described previously [27].

### 2.8. Tomato Plant Assays

#### 2.8.1. In Vitro Analysis to Determine the Effect of Exogenous Farnesol on Tomato Growth and on Expression of Plant Defense- and Development-Related Genes

Tomato seeds were sterilized, germinated and grown in Murashige and Skoog (MS) medium (Sigma-Aldrich, St Louis, MO, USA) amended with different concentrations of farnesol as described previously [28].

#### 2.8.2. Inoculation of Tomato Seeds with *Trichoderma* Spores, Plant Growth and Infection with *Botrytis cinerea* B05.10

Tomato seeds were coated with spores of T34-43b1.3 and T34-dpp1.3 strains as described previously [27]. Seeds were sown in Kekklä 50/50 substrate and incubated at 21 °C, with 50% humidity with a photoperiod of 18 h:6 h light:dark. Plants were watered with fertilizer as needed. After four weeks of growth, plants were collected and parameters such as stem thickness and stem height were recorded. Later, second level leaves of only one of the sides of the plant were infected with *B. cinerea* spores (spots of 15 μL at a concentration of 5 × 10^5^ spores/mL). The infected plants were placed in a humid chamber and incubated for four additional days using the same conditions indicated above.

Leaves from non-infected and infected plants were harvested and used for RNA extraction to analyze the expression of plant defense-related genes: *PR1b1* and *PR-P2*, related to the salicylate (SA) defense signaling pathway, and *PINI*, *PINII* and *TomLoxA*, related to the jasmonate/ethylene (JA/ET) signaling pathways [23].

#### 2.8.3. Tomato-*Trichoderma* Hydroponic Cultures for Analysis of the Effect of *dpp1* Overexpression on the Ability to Colonize Tomato Roots

Tomato hydroponic cultures were grown as described previously [19]. Once grown, roots were recovered, rinsed with sterile distilled water, and used for DNA extraction followed by quantitative PCR gene analysis. Relative amounts of Tomato *gpd* (glyceraldehyde 3′-phosphate dehydrogenase) and T34 α-actin genes were determined for this purpose.

### 2.9. Phylogenetic Analysis

Orthologs of dpp1 homologs from 34 Trichoderma species representing the breadth of known phylogenetic diversity of this genus were used for this study. The nucleotide and predicted amino acid sequences of the orthologs were aligned using MUSCLE as implemented in MEGA version 10.2.6 [29]. The alignments were then subjected to a maximum likelihood analysis (ML) using the program IQ-Tree v. 1.6.7. software [30]. Branch support was determined by bootstrap analysis using 1000 pseudoreplicates. Visualization of the phylogenetic trees was carried out using the program FigTree v1.4.4 (http://tree.bio.ed.ac.uk/, accessed on 8 November 2022). dpp1 nucleotide sequences were retrieved from the genome of the 34 Trichoderma species used in this study. Among them, 15 genomes were already available at the National Center for Biotechnology Information (NCBI) database (Table S2), while the dpp1 orthologs of the remaining 19 Trichoderma species were retrieved from genome sequences generated in previous studies [31] and were deposited at DDBJ/ENA/GenBank under the following accession numbers: OP870016 (for T. albolutescens CBS 119286), OP870017 (T. aurantioeffusum S565), OP870018 (T. balearicum CBS 133222), OP870019 (T. calamagrostidis CBS 121133), OP870020 (T. cf. fertile CBS137003), OP870015 (T. crystalligenum S38), OP870021 (T. deliquescens CBS 130572), OP870022 (T. gamsii T065), OP870023 (T. harzianum CECT 2413), OP870024 (T. margaretense S368), OP870025 (T. polysporum CBS 111723), OP870026 (T. protrudens CBS 121320), OP870027 (T. psychrophilum S647), OP870028 (T. rhododendri CBS 119288), OP870029 (T. rodmanii CBS 121553), OP870030 (T. rubi CBS 127380), OP870031 (T. stromaticum CBS 101729), OP870032 (T. taxi TUCIM 2377), OP870033 (T. turrialbense CBS 112455).

## 3. Results

### 3.1. Identification of T. harzianum Homologs of DPP1/LPP1

BLASTn analysis using the *S. cerevisiae* pyrophosphatase genes *DPP1* and *LPP1* as query sequences revealed the presence of a single homolog designated *dpp1* of these genes in the genome sequence of *T. harzianum* strain CECT2413 (T34). In BLASTp analysis using the deduced amino acid sequence of T34 *dpp1* as a query sequence against the GenBank nonredundant database of fungal sequences, the highest BLASTp scores were for putative diacylglycerol diphosphate phosphatases from other *Trichoderma* species, with percentages of identity ranging from 82% to 99% (E values = 1 × 10^−161^ to 0.0) for homologs from *T. arundinaceum* and *T. lentiforme*, respectively.

### 3.2. Effect of Overexpression of T. harzianum dpp1 on Fungal Growth

Eight phleomycin-resistant transformants were recovered following the transformation of T34 protoplasts with plasmid pTCdpp1-b1 (Figure 2A). In an initial PCR analysis using oligonucleotides TADIR2 and Th-dpp1-E4-1R (Table S1), five of the transformants yielded the 1235-bp amplicon corresponding to the *tss1* promoter and *dpp1* gene in the overexpression construct.

Genomic DNAs of the five transformants were digested with *Eco*RI and analyzed by Southern blot, using as a probe a 622 bp internal fragment to *T. harzianum dpp1*, which was PCR amplified using oligonucleotides Th-dpp1-E4-1F/Th-dpp1-E4-3R (Table S1). The same membrane was used to hybridize with a 201 bp probe corresponding to the *ble* gene, conferring phleomycin/bleomycin resistance, to confirm the presence of this gene in the genome of the isolated transformant. The *ble* probe was obtained by PCR amplification using oligonucleotides Phleo-3/Phleo-4 (Table S1) from pJL43b1 plasmid [20]. All five PCR positive transformants gave the expected Southern hybridization 2341 bp band with the 622 bp internal probe to *dpp1* gene (Figure 2B) and with the 201 bp internal to *ble* gene.

### 3.3. RT-qPCR Analysis of T. harzianum Terpene-Related Gene Expression

These studies were carried out using RNAs purified from 96 h PDB grown mycelia.

#### 3.3.1. Expression of *dpp1* in T34 Transformants with Plasmid pTCdpp1-b1

**Figure 3:**
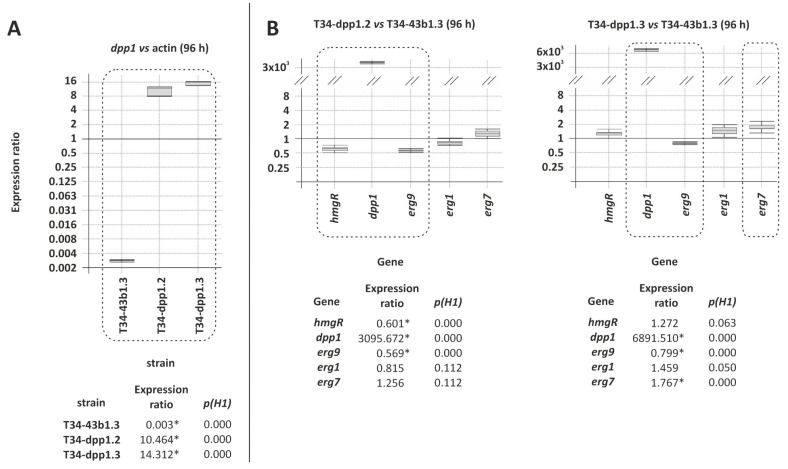
qPCR analysis from 96 h PDB grown mycelia to assess the transcription level of: (**A**) *dpp1* gene in the control strain, T34-43b1.3, that was generated from *T. harzianum* CECT 2413 by transformation with the empty expression vector used for *dpp1* overexpression, and in two *dpp1*-overexpressing transformants T34-dpp1.2 and T34-dpp1.3, relative to the expression of actin gene, used as an internal control (housekeeping). (**B**) Five genes involved in the terpene biosynthetic pathway in the two selected transformants compared versus expression in T34-43b.1. In the graphic representation, those statistically significant differential expression data (*p* < 0.05) are squared with dashed lines and numeric values were labeled with an asterisk (*) at the bottom of each graphic.

Expression of *dpp1* was almost undetectable in the wild-type strain (T34-43b1.3) reaching a relative level of 0.003 (*p* = 0.000) fold versus the level observed for the actin gene, the latter being a housekeeping gene used as a reference in the qPCR analyses (Figure 3A). Furthermore, the expression of this gene was strongly increased in all of the strains isolated by transformation with plasmid pTCdpp1-b1, being T34-dpp1.2 and T34-dpp1.3 those with the greatest up-regulation, reaching relative levels of 10.464 (*p* = 0.000) and 14.312 (*p* = 0.000) fold versus actin gene, respectively (Figure 3A). Then, these two transformants were selected for further studies. In addition, when the expression was compared versus the control strain, T34-43b1, levels reached ratios of 3095.7 (*p* = 0.000) and 6891.5 (*p* = 0.000) fold for T34-dpp1.2 and T34-dpp1.3, respectively (Figure 3B).

#### 3.3.2. Effect of *dpp1* Overexpression on the Transcription Level of Other Terpene Biosynthetic Genes

The expression of *hmgR*, encoding for the hydroxymethylglutaryl-CoA reductase, *dpp1* (farnesyl pyrophosphatase), *erg9* (squalene synthase), *erg1* (squalene epoxidase), and *erg7* (oxidosqualene cyclase) was also determined by qPCR. Results indicated that the level of expression of other terpene-related genes in these two selected *dpp1*-overexpressing transformants was only slightly affected, with non-statistically significant or with expression ratios between 0.5 and 2-fold in all cases, in comparison with the control strain, T34-43b.1 (Figure 3B).

### 3.4. Metabolomic Characterization of the T. harzianum dpp1-Overexpressed Transformants

#### 3.4.1. Production of Farnesol by *dpp1*-Overexpressed Transformants

**Figure 4:**
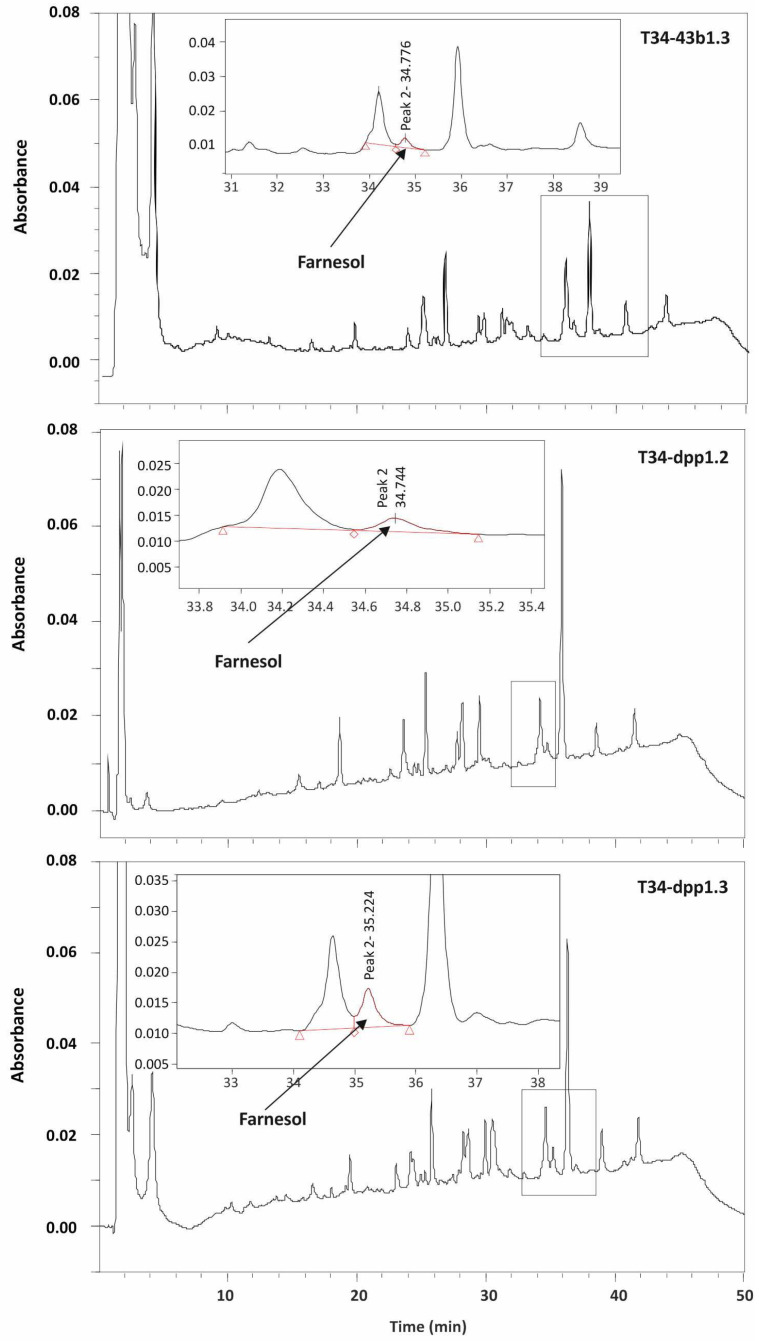
HPLC chromatograms showing levels of farnesol detected in mycelia of strains T34-43b.1 (**upper panel**), T34-dpp1.2 (**middle panel**) and T34-dpp1.3 (**bottom panel**). Farnesol levels were deduced from the area of the farnesol peak using a calibration curve performed with multiple known concentrations of pure farnesol.

Production was determined from cultures grown for 24 h. HPLC analyses revealed that the overexpression of *dpp1* increased levels of farnesol in transformant T34-dpp1.3 by 868% and 216%, in culture broth and mycelia extracts, respectively, compared to the levels observed in the same samples of the control strain T34-43b1.3 (Table 1 and Table 2) (Figure 4 and Figure S1). Despite these results, the observed differences do not correspond with the drastic relative increase in expression of the *dpp1* gene in the two overexpressing transformants. 

Furthermore, the levels of farnesol were much higher in mycelia than in culture broths, indicating that this compound is mainly accumulated intracellularly (Table 1 and Table 2).

#### 3.4.2. Production of Squalene and Ergosterol in *dpp1*-Overexpressing Transformants

**Figure 5:**
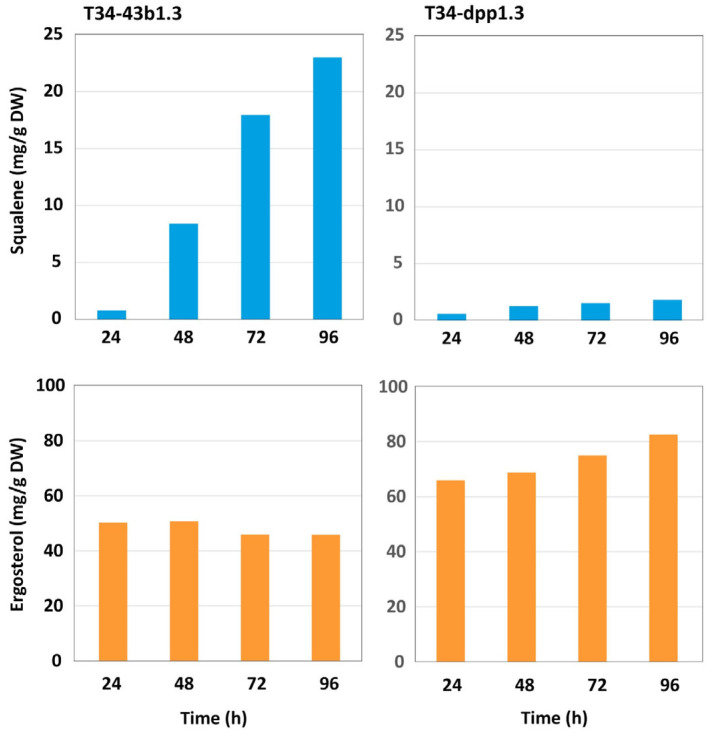
Levels of squalene (**upper panels**) and ergosterol (**bottom panels**) produced in mycelia of strains T34-43b1.3 (**left panels**) and T34-dpp1.3 (**right panels**) grown in minimal medium for 24 h to 96 h. mg/g DW = milligrams of squalene or ergosterol per gram of freeze-dried mycelia.

Production was determined from mycelia grown for 24 to 96 h. In 96 h mycelia, overexpression of the *dpp1* gene resulted in a 92% decrease in squalene levels and an 80% increase in ergosterol levels, compared versus the control strain (Figure 5). These data indicate that overexpression of the *dpp1* gene, and the subsequent increase in the level farnesol production, resulted in a drastic alteration in the level of the other terpenic compounds, which shows the important role of Dpp1 as a regulator of the intracellular FDP levels.

### 3.5. Effect of External Addition of Farnesol on the Squalene-Ergosterol Levels in T34-43b1.3 versus T34-dpp1.3

Strains T34-43b1.3 and T34-dpp1.3 were grown for 96 h in liquid minimal medium amended with 5 μM, 25 μM and 100 μM of farnesol. Mycelia were then collected by filtration, and the concentrations of squalene and ergosterol were quantified. The production of squalene was lower in the T34-dpp1.3 transformant than in the control T34-43b1.3 at all farnesol concentrations. For each strain, there were small but non-statistically significant differences in squalene levels produced at the three farnesol concentrations used (Figure S2). The levels of ergosterol produced by the *dpp1*-overexpressed and wild-type strains were similar in all concentrations used except in 25 μM farnesol treatment, where the overexpression strain produced 18.7% more ergosterol than the wild-type strain.

When the expression of terpene biosynthetic genes was analyzed in T34-dpp1.3 transformant versus the control T34-43b1.3 strain, in the presence of 100 μM farnesol, no significant differences were observed compared to the levels detected in the absence of farnesol (Figure S3). Furthermore, the pattern of expression in MMT (96 h) is clearly different from that observed in PDB (96 h) (see Figure 3). In MMT, at 96 h, all the terpene genes were down-regulated in the T34-dpp1.3 overexpressed transformant compared to T34-43b1.3, being especially marked the down-regulation of the *erg7* gene observed both with and without (woF) farnesol (Figure S3).

These data indicated that exogenous farnesol does not significantly affect the level of transcription of the key terpene genes analyzed. The differences observed in the production of ergosterol in the absence of farnesol almost disappeared when exogenous farnesol was added to the media, indicating that the added farnesol might modify the regulatory network of the farnesol–ergosterol pathways (Figure S3).

### 3.6. Importance of Farnesol in the Regulation of Circadian Cycle-Related Genes in T. harzianum

**Figure 6:**
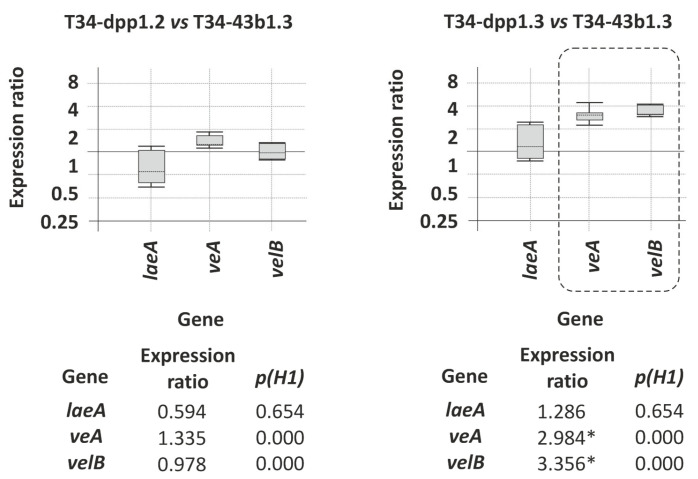
qPCR analysis of relative expression levels of genes related to regulation of circadian cycle: *laeA*, *veA* and *velB*, in transformants T34-dpp1.2 and T34-dpp1.3 versus levels observed in the control strain T34-43b1.3. In the graphic representation, those statistically significant differential expression data (*p* < 0.05) are squared with dashed lines and numeric values were labeled with an asterisk (*) at the bottom of each graphic.

The expression of *laeA*, *veA* and *velB* genes was analyzed by qPCR from 96 h PDB grown mycelia. LaeA/LAE1 (loss of *aflR* expression A) is a nuclear methyltransferase-domain containing protein that interacts with VeA and VelB to form the VELVET heterotrimeric complex. This complex synchronizes secondary metabolism and fungal development in relation to the light levels [32]. Expression of these three genes did not significantly differ between transformant T34-dpp1.2 and the control strain T34-43b1.3 (Figure 6). However, the expression of *veA* and *velB* was significantly up-regulated in transformant T34-dpp1.3 compared to T34-43b1.3, with relative expression levels that were 2.984 (*p* = 0.000) and 3.356 (*p* = 0.000) fold higher in that *dpp1*-overexpressing transformant, respectively (Figure 6). Considering that the expression level of *dpp1* and production of farnesol by transformant T34-dpp1.3 were significantly higher than in T34-dpp1.2, these data indicate that the increase in the production of farnesol affected the expression of genes involved in the regulation of circadian cycle, which agrees with the role of farnesol as a key molecule in maintaining fungal homeostasis. However, these data are not conclusive and would require a more detailed analysis in the future.

### 3.7. Effect of the Increase in Farnesol Production on the Fungal Phenotype

*T. harzianum* wild-type strain and the two *dpp1*-overexpressing transformants were grown in MEA medium containing farnesol (50 μM, 100 μM and 1000 μM). After incubation for 7 days at 28 °C, no differences in growth were observed between the wild type and the two *dpp1* overexpressing transformants (Figure S4). Furthermore, the growth of all three strains analyzed was inhibited at farnesol concentrations higher than 50 μM (Figure S4), which is a similar effect to the inhibition of yeast growth observed in the current study, i.e., *C. albicans* and *S. cerevisiae*, when increasing concentrations of farnesol were used (Figure S5).

As indicated above, the highest level of *dpp1* expression coincided with higher levels of farnesol production in transformant T34-dpp1.3. However, those farnesol levels could be affected by phosphorylation of the produced farnesol, e.g., by the activity of the farnesol kinase enzyme, which has not yet been identified but likely occurs in *T. harizanum*.

In addition, an analysis of the phenotypic characteristics was performed by growing the control strain T34-43b1.3 and the two *dpp1*-overexpressing transformants T34-dpp1.2 and T34-dpp1.3, in five different solid media to compare colony shape, sporulation rate, pigmentation, and growth rate. The media used in this study were OA, CMA, PDA, SNA and MBA. As result, after 14 days of growth at 28 °C, no differences were found between the three species analyzed (Figure S6).

### 3.8. Effect of dpp1 Overexpression on Trichoderma–Plant Interactions

#### 3.8.1. Effect of Exogenous Farnesol on Tomato Plants

**Figure 7:**
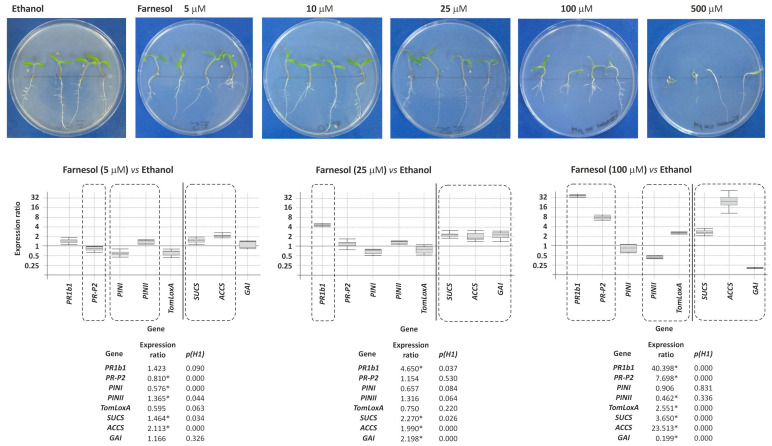
Analysis of the effect of different farnesol concentrations on the growth of tomato plantlets (**Upper panels**) and the relative expression of five tomato defense-related genes: *PR1b1* and *PR-P2*, related to the salicylic acid signaling pathway, and *PINI*, *PINII* and *TomLoxA* related to the jasmonate/ethylene signaling pathways, and three development-related genes: *SUCS*, *ACCS*, and *GAI*, as determined by quantitative PCR (**Lower panels**). In the graphic representation, those statistically significant differential expression data (*p* < 0.05) are squared with dashed lines and numeric values were labeled with an asterisk (*) at the bottom of each graphic.

Tomato plantlets were grown in 150 mm Petri dishes containing 50 mL of MS medium amended with 5, 10, 25, 100 and 200 μM farnesol. Visible effects on plant phenotype were observed only at 100 and 200 μM farnesol. However, these concentrations were well above the physiological levels. The effects of exogenous farnesol on the expression of genes involved in plant defense (5 genes) and development (3 genes) were also examined by growing tomato plantlets as described above on 0, 5, 25 and 100 μM farnesol. The highest farnesol concentration induced the up-regulation of the plant defense-related genes *PR1b1* and *PR-P2*, which belong to the salicylic signaling pathway, but lower farnesol concentrations did not. Exogenous farnesol also induces a significant up-regulation of the plant defense gene *TomLoxA* [2.551 (*p* = 0.000) fold] at 100 μM but not at the lower concentrations (Figure 7). The expression of other plant defense-related genes (*PINI* and *PINII*) was not affected by exogenous farnesol. Finally, the expression of two of the plant development-related genes, such as *SUCS* and *ACCS*, was up-regulated at all three concentrations analyzed, while *GAI* was slightly up-regulated at 25 μM farnesol and strongly downregulated at 100 μM farnesol (Figure 7).

#### 3.8.2. Effect of *dpp1* Overexpression in *T. harzianum* on Plant Development

Tomato seeds were coated with T34-43b1.3 and T34-dpp1.3 conidia and then incubated as described in the Section 2. After four weeks of incubation, growth of the resulting plants was assessed by measuring stem diameter and length. We did not observe significant differences in tomato plant growth following the treatment of seeds with the *T. harzianum* strains. Treatment with T34-43b1.3 (control strain) resulted in an average stem diameter of 8.0 ± 0.4 cm and stem length of 22.5 ± 1.9 cm (*n* = 14), whereas treatment with the *dpp1*-overexpressed strain resulted in an average stem diameter of 7.7 ± 0.6 cm, and stem length of 19.0 ± 2.2 cm (*n* = 14). These results indicate that the overexpression of *dpp1* in *T. harzianum* did not affect plant growth during the plant–fungus interaction.

#### 3.8.3. Effect of *dpp1* Overexpression on Resistance of Tomato Plants to *Botrytis cinerea*

**Figure 8:**
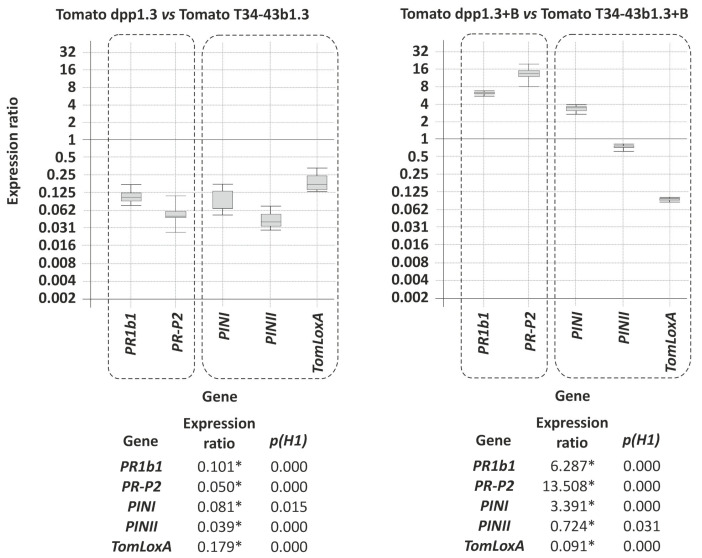
qPCR analysis of expression of five tomato defense-related genes. The relative expression of these genes has been analyzed in leaves grown from tomato seeds coated with T34-dpp1.3 versus leaves from seeds inoculated with T34-43b1.3, in both cases without *Botrytis cinerea* B05.10 infection (**left panel**), or leaves from the same plants after infection with *B. cinerea* (**right panel**). Tomato α-actin gene has been used as a housekeeping-reference gene in this study. In the graphic representation, those statistically significant differential expression data (*p* < 0.05) are squared with dashed lines and numeric values were labeled with an asterisk (*) at the bottom of each graphic.

Comparison of healthy tomato plants that had not been inoculated with *B. cinerea* but had been treated with the control strain (T34-43b1.3) or a *dpp1* overexpression strain (T34-dpp1.3) of *T. harzianum* revealed that *dpp1* overexpression caused the down-regulation of all defense-related genes examined. For example, the expression rations range from 0.050 (*p* = 0.000) and 0.179 (*p* = 0.000) fold for the *PR-P2* and *TomLoxA* genes, respectively (Figure 8). A possible explanation for these results is that increased intracellular farnesol concentrations and/or changes in squalene and ergosterol levels in the *dpp1* overexpression strain contributed to the observed down-regulation. There are previous reports that indicate the importance of ergosterol/squalene levels of *Trichoderma* on the ability of the fungus to colonize plant roots and on the regulation of the plant defense response [28].

The expression of plant defense related genes in health and *B. cinerea*-infected plants differed markedly. In infected plants, *PR1b1* and *PR-P2* genes were relatively up-regulated, with ratios of 6.3 (*p* = 0.000) and 13.5 (*p* = 0.000), respectively. In contrast, among the genes related to the JA/ET signaling pathway, only *PINI* was up-regulated [3.4 (*p* = 0.000) fold], whereas *PINII* and *TomLoxA* were down-regulated (Figure 8). The up-regulation of SA-related genes (*PR1b1, PR-P2*) has been also previously observed in response to necrotrophic pathogens or to volatile organic compounds produced by these pathogens [28]. A possible explanation for the higher level of expression of SA-related genes in T34-dpp1.3-treated plants in the presence of *B. cinerea* could be that the increased farnesol production and/or concomitant deregulation of ergosterol biosynthesis could induce a higher level of sensitization of plants against fungal plant pathogens.

#### 3.8.4. Effect of Increased Farnesol Production on Root Colonization

Roots of hydroponic grown tomato plants were inoculated with T34-43b1.3 (control strain) and T34-dpp1.3 to determine the effect of *dpp1*-overexpression in *T. harzianum* on the tomato root colonization ability. For this analysis, qPCR was used to monitor the levels of the tomato glyceraldehyde 3′-phosphate dehydrogenase gene (*gpd*) versus the *T. harzianum* α-actin gene (*act1*). The analysis revealed that the relative level of *T. harzianum act1* in roots from T34-dpp1.3 treated plants was only 52% of the level in roots of plants treated with the *T. harzianum* T34 wild-type strain. These results indicate that increased *dpp1* expression reduced the ability of *T. harzianum* to colonize tomato roots.

### 3.9. Effect of dpp1 Overexpression on the Trichoderma Antifungal Activity

#### 3.9.1. Antifungal Activity against *B. cinerea* B05.10 on Tomato Leaves

This analysis was performed using 96 h grown PDB culture broths from T34-43b1.3, T34-dpp1.2, and T34-dpp1.3. Two 15 μL aliquots of a *B. cinerea* spore suspension, 5 × 10^5^ spores/mL, were deposited onto tomato leaves, each aliquot at a different position in the leaf. Once the aliquots had dried, a 15 μL aliquot of a culture broth from one *T. harzianum* strain was deposited on each dried spot of *B. cinerea* spores. Leaves were then incubated on a humid atmosphere for 72 h, after which the size of any resulting lesions was measured. No significant differences were observed in the sizes of lesions resulting from the treatment of broth of the wild-type strain and the two *dpp1* overexpressing transformants (Figure S7). Thus, the overexpression of *dpp1* did not have a significant effect on the antifungal activity of *T. harzianum* against the pathogen *B. cinerea*.

#### 3.9.2. Antifungal Assays on Membranes against *R. solani*

Metabolites secreted by the three *Trichoderma* strains assayed in this study, T34-43b1.3, T34-dpp1.2, and T34-dpp1.3, did not have any significant inhibitory effect on *R. solani* growth (Figure S8). Thus, similar to the results with *B. cinerea* shown above, the overexpression of *dpp1* did not affect the antifungal activity of *T. harzianum* against the pathogen *R. solani*.

### 3.10. Phylogenetic Analysis of Trichoderma dpp1 Orthologs

**Figure 9:**
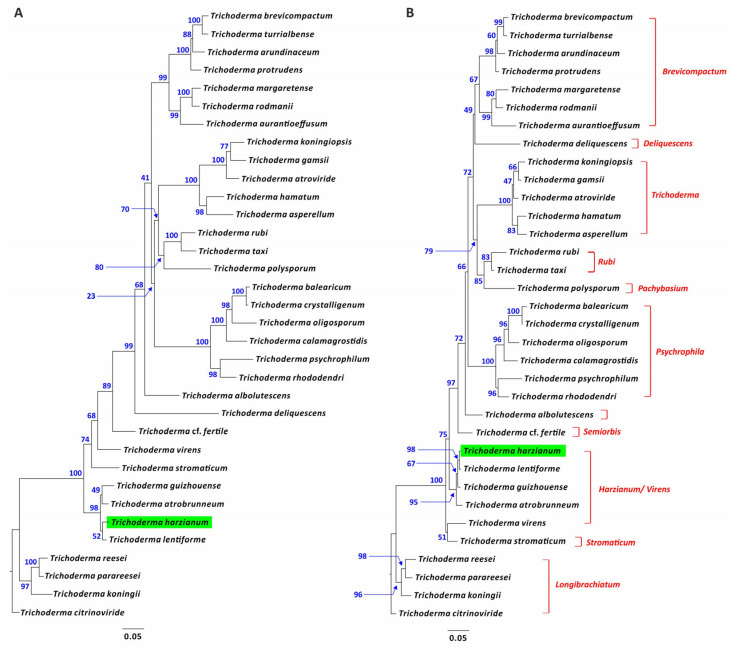
Maximum likelihood phylogenetic trees inferred from full-length *dpp1* exon (**A**) or Dpp1/DPP1 amino acid (**B**) sequences retrieved from the genome of 34 *Trichoderma* species that represent the known phylogenetic breadth of the genus. The trees were inferred as described in the Section 2. The numbers on each branch are bootstrap values derived from 1000 pseudoreplicates. The names of previously described subgeneric lineages [31,33] are indicated in red type at the right of the figure. The location of the *T. harzianum dpp1* gene and Dpp1/DPP1 protein in the trees has been highlighted with a green rectangle. The best substitution models used to generate these phylogenetic trees were determined by the IQ-Tree software as TIM3+F+I+G4 and JTTDMut+I+G4 for the nucleotide and amino acids alignments, respectively, on the basis of the optimal AICc (Akaike Information Corrected) and BIC (Bayesian Information) criteria.

The phylogenetic relationships of *dpp1* orthologs in *Trichoderma* was assessed using full-length coding or deduced amino acid sequences of orthologs from 34 *Trichoderma* species representing the known breadth of phylogenetic diversity within the genus. The topologies of the resulting nucleotide and amino acid trees were similar to one another (Figure 9) and to a previously reported species tree inferred from concatenated alignments of 20 housekeeping genes from the same set of species [31]. The presence of a *dpp1* homolog in all 34 *Trichoderma* species examined and the mirroring of the *dpp1* and species trees is consistent with *dpp1* having an essential role in primary metabolism.

## 4. Discussion

Farnesol is an isoprenoid intermediate of the mevalonate pathway produced by the dephosphorylation of FDP (Figure 1). FDP plays a central role in cell growth and differentiation; it controls the production of ubiquinone and ergosterol but also participates in the regulation of filamentation and biofilm formation [7,8].

In yeast, reductions in FDP dephosphorylation found in *dpp1-*, and also in *lpp1-*deleted strains were explained by the involvement of the corresponding proteins, Dpp1 and Lpp1, in avoiding the toxic accumulation of isoprenoid pyrophosphates, including FDP. Such accumulation could result from diverse processes, e.g., down-regulation of squalene synthase activity, which catalyzes the first committed step in ergosterol biosynthesis (i.e., conversion of FDP to squalene) [34]. Overexpression of the FDP synthase gene (*ERG20*) in a yeast mutant lacking squalene synthase activity led to a dramatic increase in the level of dolichol [35], indicating that the FDP pool should be shunted toward dolichol, but the dephosphorylation of FDP may also be important [13,17].

The purpose of the current study was to assess the effect of farnesol production on the physiology and ecology of *Trichoderma*. To do this, we identified a homolog (*dpp1*) of the yeast *DPP1* and *LPP1* genes in *T. harzianum* T34, which is a well-characterized and considered a model in the biological control studies. An initial gene expression analysis revealed that *dpp1* exhibits very low levels of expression in wild-type *T. harzianum*. This low-level expression of *dpp1* in the wild type could explain why previous attempts to silence *dpp1* expression in *Trichoderma* species were unsuccessful (Gutiérrez S. unpublished results). In subsequent analyses, introduction of an overexpression construct into T34 increased *dpp1* expression by over 1000-fold. Farnesol production was increased in the two selected transformants (T34-dpp1.2 and T34-dpp1.3) compared to the level observed in the untransformed strain (Table 1 and Table 2), with T34-dpp1.3 reaching the highest levels in agreement with the higher level of *dpp1* gene expression observed in this transformant. The increase in farnesol production in T34-dpp1.3 was accompanied by a marked decrease in squalene production and a moderate increase in ergosterol production. However, the huge increase in the level of *dpp1* gene expression does not match the comparatively moderate increases in farnesol production. This discrepancy could result from the toxicity of the increased levels of farnesol, but other factors as RNA instability could also explain these results. Furthermore, due to the reversibility of the FDP dephosphorylation reaction, farnesol can be detoxified by channeling it to other terpenoids. Indeed, we observed increased levels of ergosterol in the *dpp1* overexpression strains (Figure 5). The increased farnesol production in *dpp1* overexpressed transformants resembles the increase in FDP production observed in yeast as result of a mutation in the *ERG9* gene, which encodes the enzyme, squalene synthase, that catalyzes the first committed step in ergosterol biosynthesis from FDP [36].

The previously reported effects of farnesol on yeast morphogenesis, as well as in other processes critical for fungal survival, such as the regulation of quorum-sensing and protein prenylation, has also been observed at a transcriptomic level [37], where changes were observed in the expression of genes belonging to several functional categories, including iron transport, cell wall synthesis, drug resistance, and cell cycle progression. In the current study, overexpression of the *dpp1* gene had only minor effects on the expression of ergosterol biosynthetic genes. In contrast, *dpp1* overexpression in *T. harzianum* significantly up-regulated expression of the velvet complex genes *veA* and *velB* but not *laeA*. This heterotrimeric complex, consisting of the LaeA, VeA and VelB proteins, synchronizes secondary metabolism and fungal development in relation to the light levels [32]. Thus, the results of the current study suggest that farnesol affects fungal physiology beyond the morphogenesis and quorum-sensing regulation.

Exogenously added farnesol reduced the growth of tomato plantlets and up-regulated SA-related defense genes. This phytotoxic effect was also observed in studies of bean plants [38], where the addition of exogenous farnesol at concentrations between 10 and 100 mM caused reduced development of the root system, suggesting that these farnesol concentrations were toxic to bean plants [38]. Furthermore, a similar up-regulation of SA-related genes was also found in tomato plants inoculated with *Trichoderma* strains producing the mycotoxin harzianum A (HA), which would indicate that farnesol, similarly to HA, acts as a plant sensitizing metabolite (microbial-associated molecular patterns = MAMP or pathogen-associated molecular patterns = PAMP) [27,39]. These results suggest that exogenously added farnesol is perceived as a toxic compound by plants, and as result, they activate their response in a manner similar to how they respond to *Trichoderma* phytotoxic secondary metabolites, e.g., trichothecenes and aspinolides [27,28].

In addition to farnesol, other terpene analogs, e.g., squalene and ergosterol, have been deeply characterized regarding their importance on the interaction of *T. harzianum* with plants. Thus, studies performed using pure squalene/ergosterol at concentrations similar to those found in the *T. hazianum* T34 mycelium resulted in an up-regulation of genes related to the jasmonate/ethylene (JA/ET) signaling pathways and no effect on SA-related genes [21]. The effects observed for ergosterol/squalene contrast with those found in the present study in plants grown in media amended with farnesol or in plants inoculated with the strain T34-dpp1.3, overexpressing *dpp1* gene, in which it was observed that an increase in farnesol production resulted in an up-regulation of SA related genes, with a smaller or no effect on expression of JA/ET related genes.

The results discussed above would indicate that fungal farnesol, ergosterol and squalene play important roles in the fungal–plant interaction, acting as MAMPs or PAMPs. Thus, being their relative amounts found in a given *Trichoderma* strain essential for the detected plant response, with concentrations up-regulating SA related genes, e.g., increase in farnesol production, and others up-regulating JA/ET related genes, e.g., high ergosterol/low squalene production [21].

## 5. Conclusions

The functional characterization of *dpp1* overexpression strains of *T. harzianum* indicate that the corresponding enzyme is involved in the dephosphorylation of FDP to produce farnesol. Therefore, the *dpp1*-encoded enzyme is most likely a diacylglycerol pyrophosphatase. However, the observed levels of farnesol were much lower than those expected based on the high levels of *dpp1* gene expression reached by the transformants. These data suggest that farnesol levels are tightly regulated in order to avoid the toxic effects associated with its cellular accumulation. The increase in farnesol production was sufficient to have a marked effect on fungal physiology that was exemplified by changes in levels of squalene/ergosterol, but they do not have a significant effect on growth or in the antifungal activity of the *dpp1* overexpressing strains. Finally, the increase in farnesol production: (i) had a plant sensitization-like effect, in such a way that tomato plants inoculated with a *dpp1* overexpression transformant strain exhibited an up-regulation of plant defense genes related to the salicylate signaling pathway when a phytopathogenic fungi was present, and (ii) resulted in a reduction in the ability to colonize tomato roots compared to the wild-type strain. The results of the current study provide additional insights into the role of farnesol in physiology and environmental interactions in the biological control agent *T. harzianum*.

## Figures and Tables

**Table 1:** Quantification of farnesol from 24 h culture broths.

Sample	Concentration (μg/mL)	Specific Concentration (μg/mg protein)	% Variation
T34	0.0444	0.0037	-
T34-dpp1.2	0.0811	0.0061	+64.86%
T34-dpp1.3	0.4983	0.0358	+867.57%

**Table 2:** Quantification of farnesol from 24 h grown mycelia.

Sample	Concentration (μg/mL)	Specific Concentration (μg/mg Dry Weight)	% Variation
T34	0.6102	0.0122	-
T34-dpp1.2	0.8230	0.0165	+35.24%
T34-dpp1.3	1.9278	0.0386	+216.40%

## Data Availability

*dpp1*/Dpp1 nucleotide/amino acid sequences from the 19 *Trichoderma* species whose genome sequences were not yet available at the NCBI database were deposited at DDBJ/ENA/GenBank under the following accession numbers: OP870016 (for *dpp1*/Dpp1 of *T. albolutescens* CBS 119286), OP870017 (*T. aurantioeffusum* S565), OP870018 (*T. balearicum* CBS 133222), OP870019 (*T. calamagrostidis* CBS 121133), OP870020 (*T.* cf. *fertile* CBS137003), OP870015 (*T. crystalligenum* S38), OP870021 (*T. deliquescens* CBS 130572), OP870022 (*T. gamsii* T065), OP870023 (*T. harzianum* CECT 2413), OP870024 (*T. margaretense* S368), OP870025 (*T. polysporum* S647), OP870026 (*T. protrudens* CBS 121320), OP870027 (*T. psychrophilum* S647), OP870028 (*T. rhododendri* CBS 119288), OP870029 (*T. rodmanii* CBS 121553), OP870030 (*T. rubi* CBS 127380), OP870031 (*T. stromaticum* CBS 101729), OP870032 (*T. taxi* TUCIM 2377), OP870033 (*T. turrialbense* CBS 112455).

## Supplementary Materials

The following supporting information can be downloaded at: https://www.mdpi.com/article/10.3390/jof8121266/s1, Table S1: Oligonucleotides used in the current study; Table S2: Genomic sequences of *Trichoderma* spp. retrieved from NCBI database for their use in the phylogenetic analysis carried out in the current study; Figure S1: HPLC chromatograms showing farnesol production in culture broths; Figure S2: Level of squalene and ergosterol produced in mycelia; Figure S3: Analysis by qPCR of expression of genes involved in ergosterol biosynthetic pathway in a *dpp1* overexpressing transformant; Figure S4: Effect of farnesol on growth of wild-type strain and *dpp1*-overexpressing transformants; Figure S5: Toxicity of farnesol against *Candida albicans* and *Saccharomyces cerevisiae*; Figure S6: Effect of *dpp1* overexpression on growth in different media; Figure S7: Antifungal assay on tomato leaves of culture broths from wild-type strain and two *dpp1*-overexpressing transformants; Figure S8: Effect of the *Trichoderma* strains analyzed in the current study on growth of the phytopathogen *Rhizoctonia solani* strain R43.
