# Supplementary material for: Effect of Farnesol in Trichoderma Physiology and in Fungal–Plant Interaction
Source: J Fungi (Basel). 2022 Nov 30;8(12):1266. doi: 10.3390/jof8121266 (PMC9783820; doi:10.3390/jof8121266)
Supplement: Supplementary file 1 [file jof-08-01266-s001.zip › jof-2054358-supplementary.pdf]

Supplementary material

Effect of farnesol in *Trichoderma* physiology and in fungal-plant interaction

Rosa E. Cardoza [1], Susan P. McCormick [2], Laura Lindo [1], Sara Mayo-Prieto [3], David González-Cazón [1], Natalia Martínez-Reyes [1], Guzmán Carro-Huerga [3], Álvaro Rodríguez-González [3], Robert H. Proctor [2], Pedro A. Casquero [3] and Santiago Gutiérrez [1,*]

Table S1. Oligonucleotides used in the current study.

**Name**		**Sequence 5´-3´**	
**Construction of pTCdpp1-b1 to overexpress *T. harzianum dpp1* gene**			
Th-dpp1 5F		ATGCCTTCTGTGCGCACCG	
Th-dpp1 3R		TCAGCTTCGTCGAGTTCCTA	
**Analysis of *dpp1* overexpressed transformants, and to amplify a probe for Southern experiments**			
Th-dpp1-E4-1F		ATCCCTCTAGGCGTCCTCAT	
Th-dpp1-E4-3R		TACCTTGCTCTTCTTCGTCCC	
TADIR2		TGACCACTTCGCTGCCTATC	
Th-dpp1-E4-1R		GAGGACGTTGGGTTTGATGT	
**Amplification of *ble* gene to be used as a probe in Southern experiments**			
Phleo-3		GGTGTTGGTCGGCGTCGG	
Phleo-4		TGGGTGTGGGTGCGCGG	
**Oligos for qPCR analysis of *Trichoderma* terpene-related genes**			
**Gene**	**Oligo name**	**Sequence 5´-3´**	**Efficiency (%)**
actin	aactinF	ACTGGGACGACATGGAGAAG	92
	aactinR	GGCCTGGATGGAGACATAGA	
hmgR	Th hmgR 1F	ACGAGGAGGTCGTTGAGCTA	101
	Th hmgR 1R	TAGTGCCTGTAGGGCAGCTT	
erg1	erg1-1F	CGTCGAGACAGAGGTTGTCA	90
	erg1-1R	GCTTTCGAAACTTGGAGTCG	
erg7	Th-erg7-F	ACTGGATCAGGACCAACCAG	107
	Th-erg7-R	GCTTTCAGACCAACCACCAT	
erg9	Th-erg9-1F	GGCAGACTACGCACAAAACA	101
	Th-erg9-1R	GTTAGGCCTTCACCAACCAA	
dpp1	Th-dpp1 1F	ATCCCTCTAGGCGTCCTCAT	89
	Th-dpp1 1R	GAGGACGTTGGGTTTGATGT	
**Oligos for qPCR analysis of *Trichoderma* genes related with regulation of circadian cycle**			
**Gene**	**Oligo name**	**Sequence 5´-3´**	**Efficiency (%)**
laeA	Th-laeA 1F	AGAACAACAGGCGCTACCAT	90
	Th-laeA 1R	ATTCTGTGGGTTCTCCAACG	
veA	Th-vea 1F	CAGCTTGTACGAGACGACCA	102
	Th-vea 1R	ATGAGTCCGGGGAACTTCTT	
velB	Th-velB-E1 4F	GTCAATCAGCAACCGCAGTA	106
	Th-velB-E1 4R	GGGGGTAGTTTGCAGTGTGT	
**Oligos for qPCR analysis of tomato genes related with defense and growth**			
**Gene**	**Oligo name**	**Sequence 5´-3´**	**Efficiency (%)**
actin	Actin-F	CACCACTGCTGAACGGGAA	114
	Actin-R	GGAGCTGCTCCTGGCAGTTT	
PR1b1	PR1b1F	GCACTAAACCTAAAGAAAAATGGG	112
	PR1b1R	AAGTTGGCATCCCAAGACATA	
PR-P2	PR-P2F	GGAACAGGAACACAAGAAACAGTGA	113
	PR-P2R	CCCAATCCATTAGTGTCCAATCG	
PINI	PINIF	TGAAACTCTCATGGCACGAAAAG	108
	PINIR	GGCCACATTTGTTTTCCTTCG	

PINII	PINIIF	GGCCAAATGCTTGCACCTTT	114
	PINIIR	CGTGGTACATCCGGTGGGATA	
TomLoxA	TomLoxAF	TGAACCATGGTGGGCTGAAA	95
	TomLoxAR	CTGCCCGAAATTGACTGCTG	
ACCS	ACCSF	TGAGTTGGTGAACCATGGAA	104
	ACCSR	GCTTGAACAGCCTCAAGTCC	
SUCS	SUCSF	ATGAACCGAGTGAGGAATGG	106
	SUCSR	GCTGGACCACCGTGATTAGT	
GAI	GAIF	ACCTCCGGTGAACAATCAAG	92
	GAIR	GAACGCATTTGAACCCAGAT	

Table S2. Genomic sequences of *Trichoderma* spp. that were retrieved from the National Center for Biotechnology Information (NCBI) database for their use in the phylogenetic analysis carried out in the current study [31].

Species	GenBank accession number	Reference
Trichoderma asperellum CBS 433.97	GCF_003025105.1	Druzhinina, I.S.; Chenthamara, K.; Zhang, J.; et al. Massive lateral transfer of genes encoding plant cell wall-degrading enzymes to the mycoparasitic fungus *Trichoderma* from its plant-associated hosts. *PLoS Genet*. **2018**. 14, e1007322. doi: 10.1371/journal.pgen.1007322.
Trichoderma citrinoviride TUCIM 6016	GCF_003025115.1	
Trichoderma guizhouense NJAU 4742	GCA_002022785.1	
Trichoderma atroviride IMI 206040	GCF_000171015.1	Kubicek, C.P.; Herrera-Estrella, A.; Seidl-Seiboth, V.; et al.. Comparative genome sequence analysis underscores mycoparasitism as the ancestral life style of *Trichoderma*. *Genome Biol*. **2011**. 12, R40. doi: 10.1186/gb-2011-12-4-r40
Trichoderma virens Gv29-8	GCF_000170995.1	
Trichoderma hamatum GD12	GCA_000331835.2	Studholme. D.J.; Harris, B.; Le Cocq, K.; et al. Investigating the beneficial traits of *Trichoderma hamatum* GD12 for sustainable agriculture-insights from genomics. *Front. Plant Sci.* **2013**. 4,258. doi: 10.3389/fpls.2013.00258.
Trichoderma arundinaceum IBT 40837	GCA_003012105.1	Proctor, R.H.; McCormick, S.P.; Kim, H.S.; et al. Evolution of structural diversity of trichothecenes, a family of toxins produced by plant pathogenic and entomopathogenic fungi. *PLoS Pathog*. **2018**. 14, e1006946. doi: 10.1371/journal.ppat.1006946
Trichoderma brevicompactum IBT 40841	GCA_003012085.1	
Trichoderma atrobrunneum ITEM 908	GCA_003439915.1	Fanelli, F.; Liuzzi, V.C.; Logrieco, A.F.; et al. Genomic characterization of *Trichoderma atrobrunneum* (*T. harzianum* species complex) ITEM 908: insight into the genetic endowment of a multi-target biocontrol strain. *BMC Genomics*. **2018**. 19: 662. doi: 10.1186/s12864-018-5049-3
Trichoderma koningii JCM 1883	GCA_001950475.1	Manabe, R.; Endoh, R.; Uzuhashi, S.; et al. *Trichoderma koningii* strain JCM 1883, whole genome shotgun sequencing project. **2016.** Direct submission.
Trichoderma koningiopsis P0S7	GCA_002246995.1	Castrillo, M.L.; Bich, G.A.; Modenutti, C.P.; et al. First whole-genome shotgun sequence of a promising cellulase secretor, *Trichoderma koningiopsis* strain POS7. *Genome Announc*. **2017**. 5: e00823-17. doi: 10.1128/genomeA.00823-17
Trichoderma lentiforme CFAM-422	GCA_011066345.1	Steindorff, A.S.; Formighieri, E.F.; Midorikawa, G.E.O.; et al.. *Trichoderrma lentiforme* strain CFAM-422, whole genome shotgun sequence project. **2018**. Direct submission.
Trichoderma oligosporum CGMCC 3.17527	GCA_015266385.1	Wang, C.; Zeng, Z.; Zhuang, W.; Comparative molecular evolution of chitinases in Ascomycota with emphasis on mycoparasitism lifestyle. **2020**. Direct submission.
Trichoderma parareesei CBS125925	GCA_001050175.1	Yang, D.; Pomraning, K.; Kopchinskiy, A.; et al. Genome sequence and annotation of *Trichoderma parareesei*, the ancestor of the cellulase producer *Trichoderma reesei*. *Genome Announc*. 2017. 3, e00885. doi: 10.1128/genomeA.00885-15
Trichoderma reesei QM6a	GCF_000167675.1	Li, W.C.; Huang, C.H.; Chen, C.L.; et al.*Trichoderma reesei* complete genome sequence, repeat-induced point mutation, and partitioning of CAZyme gene clusters. *Biotechnol Biofuels*. **2017**. 10, 170. doi: 10.1186/s13068-017-0825-x

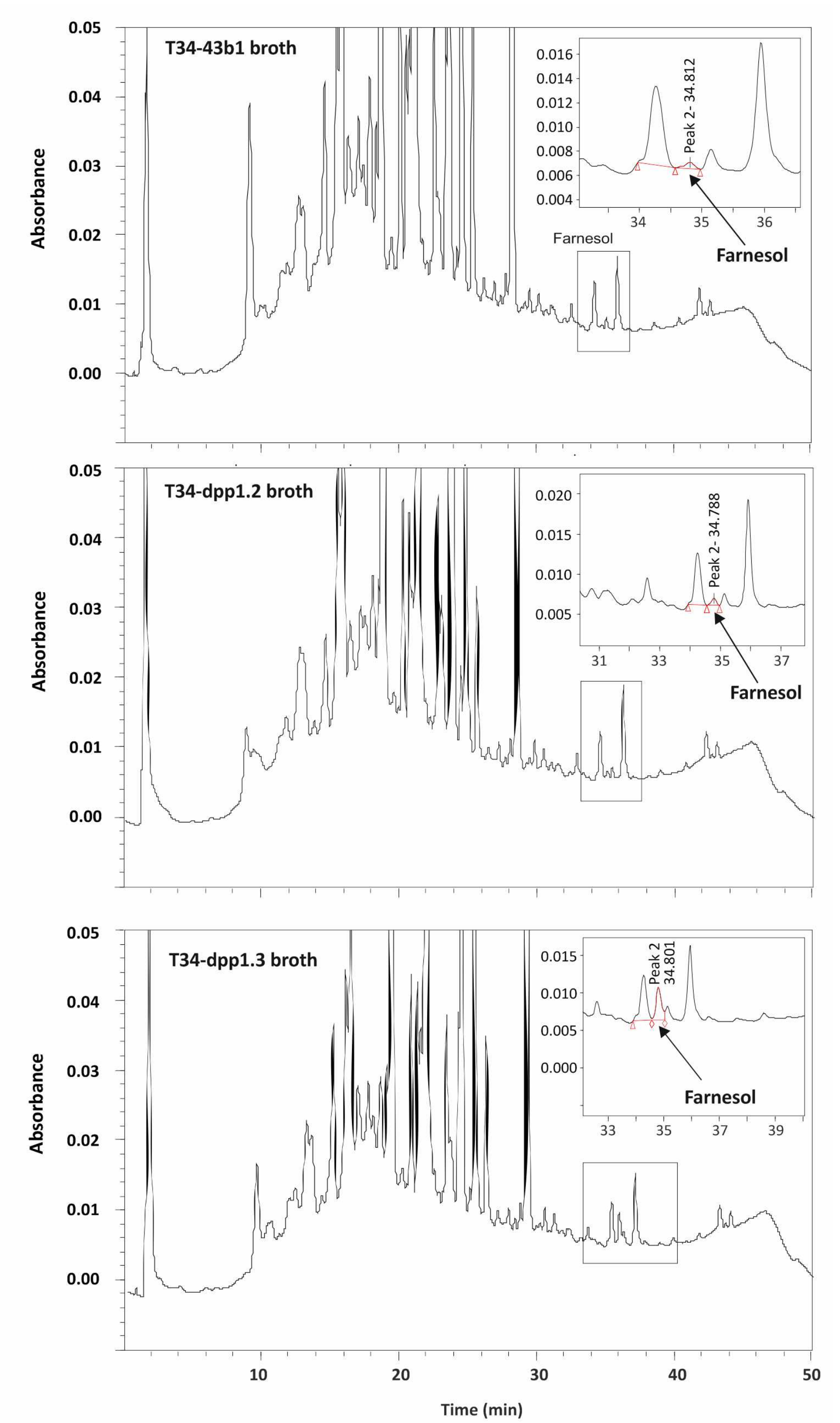

Figure S1. HPLC chromatograms showing the peaks corresponding to the farnesol production detected in 96 h culture broths of T34-43b.1 (**upper panel**), T34-dpp1.2 (**middle panel**) and T34-dpp1.3 (**bottom panel**) strains. Production of farnesol was deduced from the area of the farnesol peak using a calibration curve performed with different known concentrations of pure farnesol.

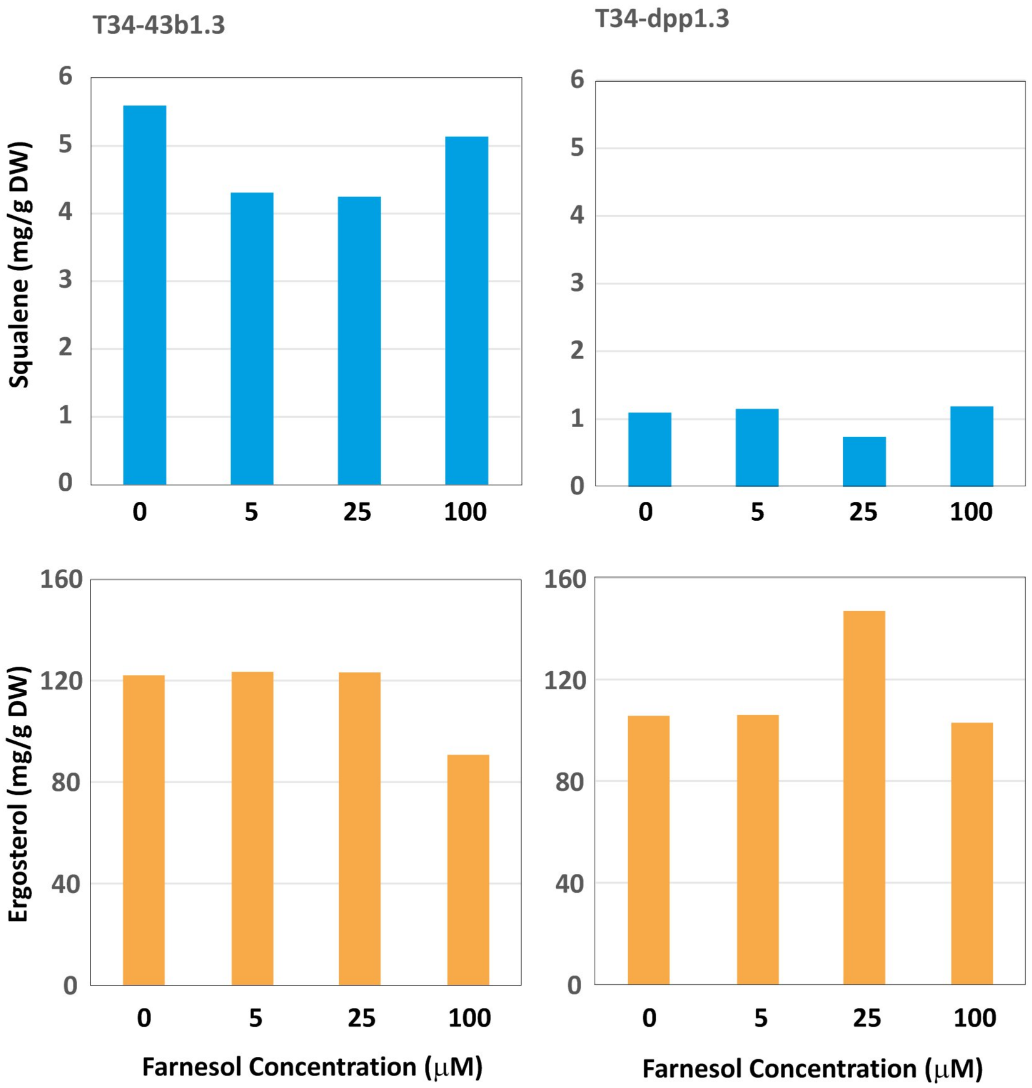

Figure S2. Level of squalene (**upper panels**) and ergosterol (**bottom panels**) produced in mycelia of strains T34-43b1.3 (**left panels**) and T34-dpp1.3 (**right panels**) grown for 96 h at 28 °C in minimal medium amended with farnesol at 0, 5, 25 or 100 μM. mg/g DW= milligrams of squalene or ergosterol per gram of freeze-dried mycelia.

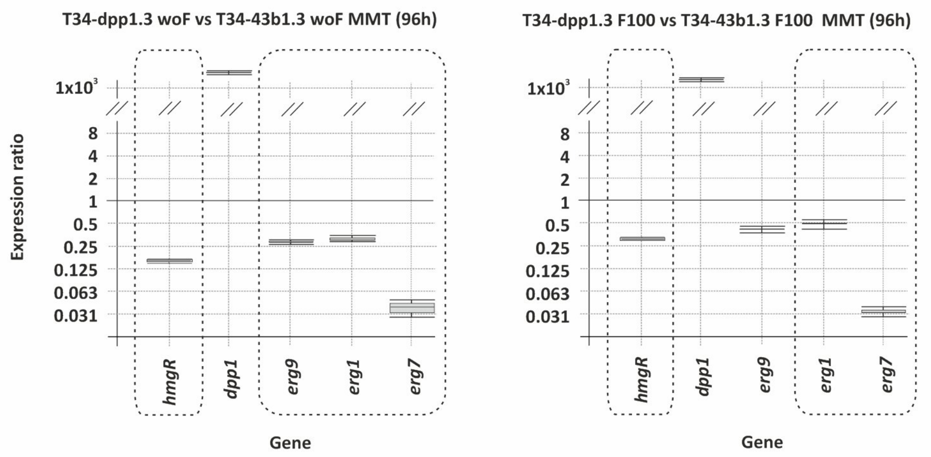

Gene	Expression ratio	p(H1)
hmgR	0.161*	0.044
dpp1	1813.401	0.066
erg9	0.283*	0.018
erg1	0.315*	0.000
erg7	0.039*	0.000

Gene	Expression ratio	p(H1)
hmgR	0.314	0.051
dpp1	1421.602*	0.000
erg9	0.415	0.103
erg1	0.484*	0.000
erg7	0.034*	0.000

Figure S3. Analysis by qPCR of relative expression levels of five genes involved in ergosterol biosynthetic pathway in the transformant T34-dpp1.3 versus expression in the control strain T34-43b1.3. The study was carried out from mycelia grown for 96 h in minimal medium (MMT) without farnesol (**left panel**) and amended with farnesol 100 µM (F100) (**right panel**). The data analysis and the graphic representation were performed as described in the legend to **Fig. 3.**

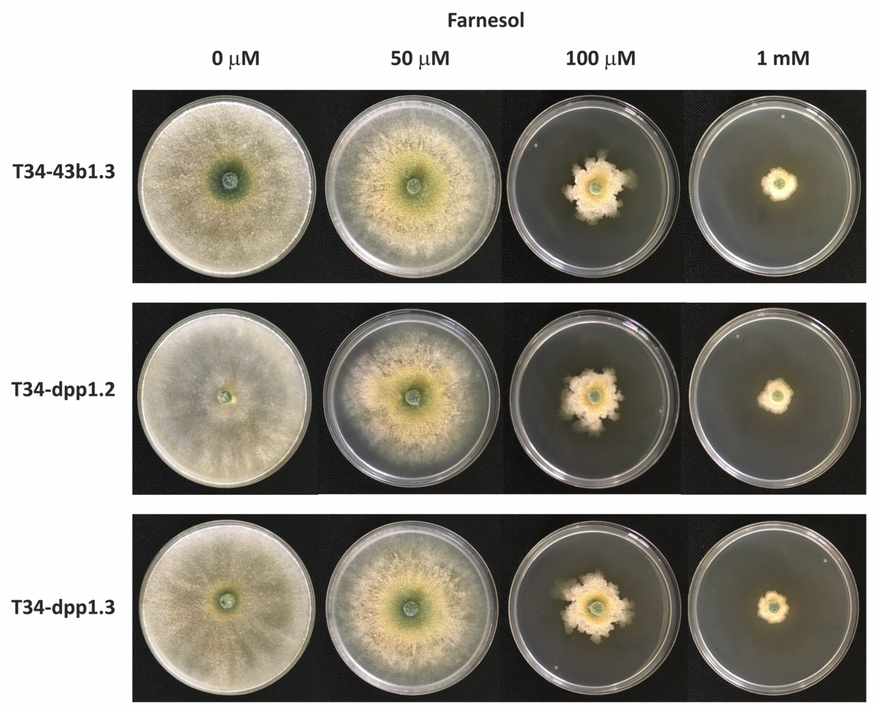

Figure S4. Effect of farnesol on growth of wild-type strain (T34-43b1.3) and the two *dpp1*-overexpressing transformants selected in the present work (T34-dpp1.2 and T34-dpp1.3). The plates were incubated for 7 days at 28 °C. Left panels correspond to the control condition without external addition of farnesol.

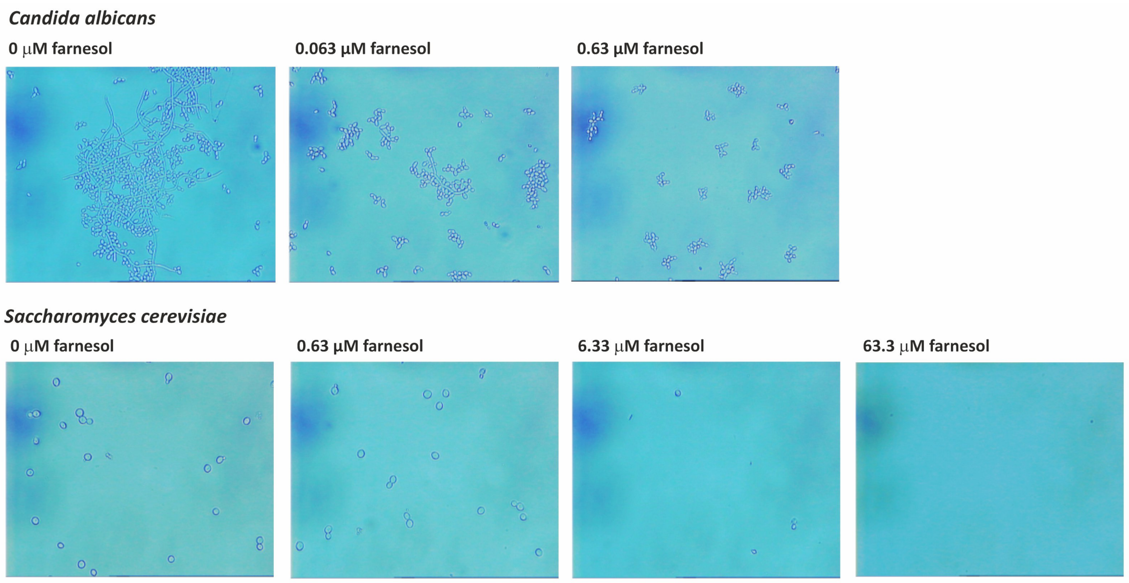

Figure S5. Toxicity of farnesol against *Candida albicans* **(upper panel**), and *Saccharomyces cerevisiae* (**bottom panel**).

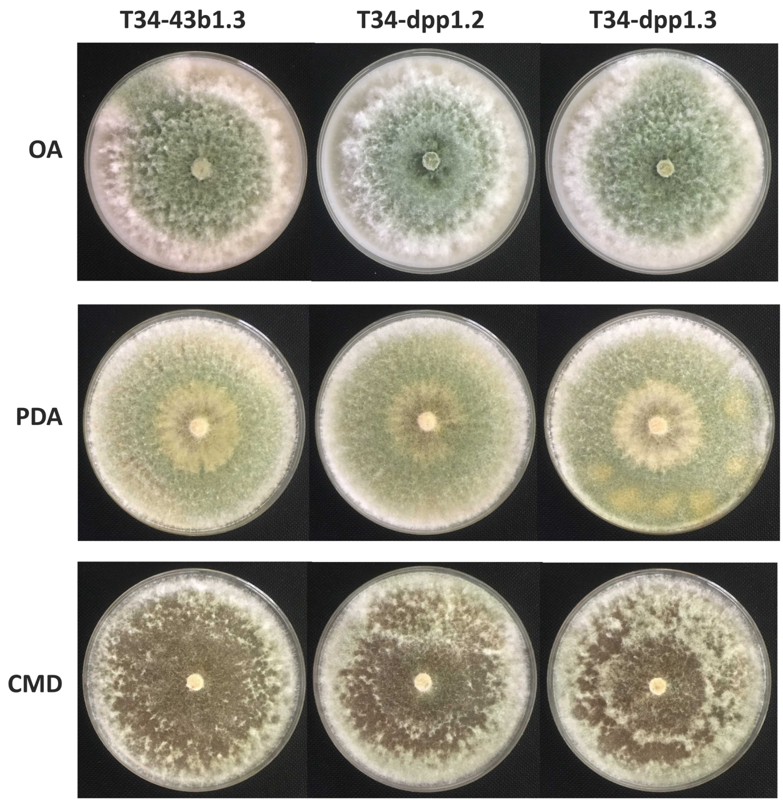

Figure S6. Effect of *dpp1* overexpression on growth in different media. Strains were grown for 14 days at 28 °C.

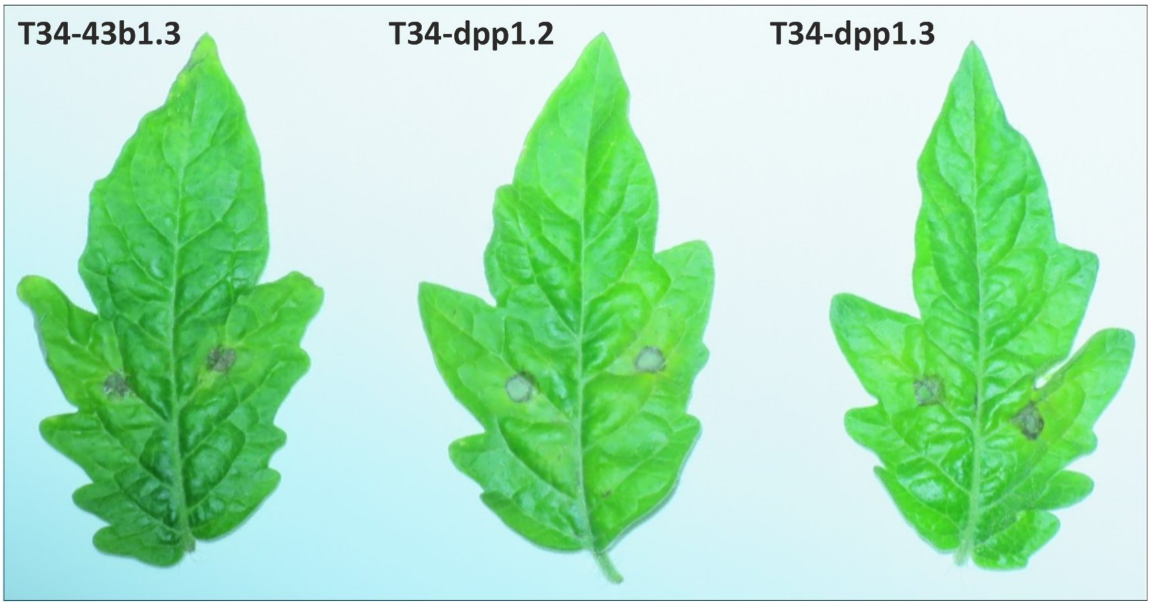

Figure S7. Antifungal assay on tomato leaves of 96 h PDB culture broths from T34-43b1.3 (control strain) and two *dpp1*-overexpressing transformants, against *Botrytis cinerea* B05.10.

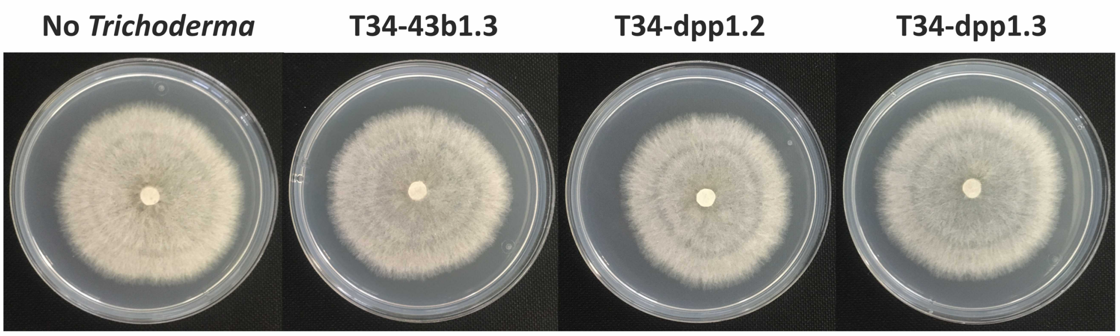

Figure S8. Effect of *T. harzianum* strains T34-43b1.3 (control strain), T34-dpp1.2 and T34-dpp1.3 (*dpp1*-overexpressing transformants) on growth of *R. solani* strain R43 in an antifungal membrane assay. Plate at the left corresponds to a control experiment in which no *Trichoderma* plug was placed on the plate before removal the cellophane membrane. Plates were incubated at 28 °C for four days after placement of the *R. solani* plug.
